# Parkinson's Disease-Induced Zebrafish Models: Focussing on Oxidative Stress Implications and Sleep Processes

**DOI:** 10.1155/2020/1370837

**Published:** 2020-08-18

**Authors:** Madalina-Andreea Robea, Ioana-Miruna Balmus, Alin Ciobica, Stefan Strungaru, Gabriel Plavan, Lucian Dragos Gorgan, Alexandra Savuca, Mircea Nicoara

**Affiliations:** ^1^Department of Biology, Faculty of Biology, “Alexandru Ioan Cuza” University of Iasi, Carol I Avenue, No. 20A, Iasi, Romania; ^2^Department of Interdisciplinary Research in Science, “Alexandru Ioan Cuza” University of Iasi, Carol I Avenue, No. 11, Iasi, Romania; ^3^Department of Research, Faculty of Biology, “Alexandru Ioan Cuza” University of Iasi, Carol I Avenue, No. 20A, Iasi, Romania

## Abstract

The complex yet not fully understood pathophysiology of Parkinson's disease includes an important molecular component consisting of oxidative status changes, thus leading to oxidative stress occurrence. While no particular evidence has been reported that describes the relationship between oxidative stress and the molecular mechanisms behind Parkinson's disease development, animal model studies has shown that oxidative stress induction could modulate Parkinson's disease symptomatology. Despite the inability to perfectly replicate human disease in animals and despite that Parkinson's disease has not been reported in any animal species, animal modeling is one of the most important tools in understanding the complex mechanisms of human disorders. In this way, this study is aimed at detailing this particular relationship and describing the molecular mechanisms underlying Parkinson's disease in animal models, focusing on the potential advantages and disadvantages of zebrafish in this context. The information relevant to this topic was gathered using major scientific database research (PubMed, Google Scholar, Web of Science, and Scopus) based on related keywords and inclusion criteria. Thus, it was observed that oxidative stress possesses an important role in Parkinson's disease as shown by numerous animal model studies, many of which are based on rodent experimental models. However, an emerging impact of the zebrafish model was observed in the research of Parkinson's disease pathological mechanisms with regard to disease development factors and the cause-effect relationship between oxidative stress and comorbidities (such as depression, hyposmia, fatigue, sleep disturbances, and cognitive deficits) and also with regard to the pharmacological potential of antioxidant molecules in Parkinson's disease treatment.

## 1. Introduction

Oxidative stress (OS) greatly impacts the human body leading to well-known pathologies, such as diabetes, atherosclerosis, Alzheimer's disease, and Parkinson's disease (PD) [1, 2]. The main cause of OS occurrence is due to the imbalance between reactive oxygen species (ROS) production and the ability of the biological systems to transform ROS into harmless oxygen species (such as water), or to detoxify the intermediate metabolites or to repair the oxidative damage [1, 3].

The concept of OS implication in mental illness was previously described [4, 5]; however, it is controversial. It is generally known that, with several exceptions, the occurrence of mental illnesses consists of the interaction between genetic or developmental events and environmental factors [6, 7]. Based on the context that mitochondrial dysfunction is facilitated by several different mechanisms and also on the fact that high levels of ROS are needed in the nervous system due to autophagy and mitophagy functions [8], OS occurrence in both the normal and pathological brain functions is currently accepted as a normal yet controlled mechanism.

**Figure 1:**
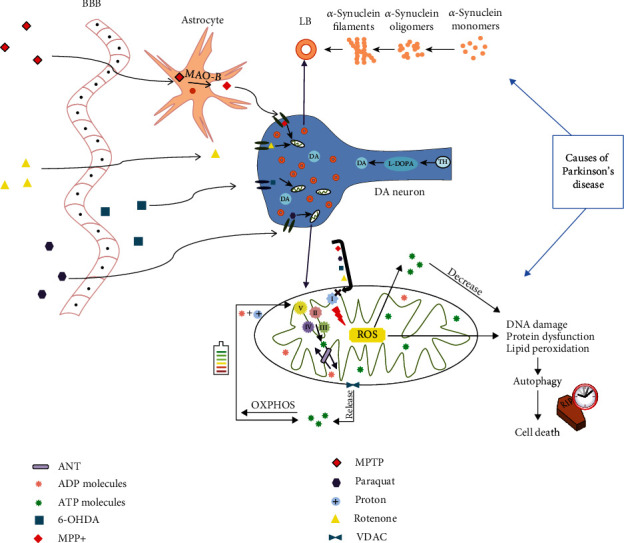
Parkinson's disease mechanism of action in the central nervous system and the pharmacokinetic effects of several agents that induce Parkinson-like symptoms. MPTP (1-methyl-4-phenyl-1,2,3,6-tetrahydropyridine) crosses the blood-brain barrier to be metabolized in 1-methyl-4-phenylpyridinium (MPP+) by monoamine oxidase B in the astrocytes. Afterwards, the transportation system of the synaptic cleft assists the intraneuronal MPP+ transfer and transports it further into the mitochondria where it impairs the mitochondrial respiration chain leading to reactive oxygen species production and dopaminergic neuron loss [21]. Similar to MPTP, paraquat could increase reactive oxygen species production, but in contrast to MPTP, it could lead to Lewy body (LB) formation [22]. 6-Hydroxidopamine could also enter the dopaminergic neurons and lead to reactive oxygen species production in the absence of the Lewy body inclusions [23]. Following diffusion to intraneuronal space, rotenone inhibits mitochondrial complex I and promotes the formation of Lewy body inclusions [22, 23]. Abbreviations: 6-OHDA—6-hydroxydopamine; ADP—adenosine diphosphate; ANT—adenine nucleotide translocase; ATP—adenosine triphosphate; BBB—blood-brain barrier; DA—dopamine; LB—Lewy bodies; L-DOPA—levodopa; MAO-B—monoamine oxidase B; MPP+—1-methyl-4-phenylpyridinium; MPTP—1-methyl-4-phenyl-1,2,3,6-tetrahydropyridine; OXPHOS—oxidative phosphorylation; ROS—reactive oxygen species; TH—tyrosine; VDAC—voltage-dependent anion channel.

Considering all these aspects, PD can be defined as a complex neuropsychiatric disorder occurring mostly in elders, which is commonly characterized by dopaminergic system malfunction leading to muscular tonus loss [9]. The exact cause of PD is unknown, but several hypotheses point at genetic inheritance, drugs, and environmental factors, such as pesticides, heavy metals, cigarette smoking, and caffeine [10, 11]. The administration of several chemical compounds can influence the entire cell metabolism leading to a cascade of events as shown in Figure 1.

The emerging use of the zebrafish model in neurological and neurodegenerative human diseases (such as PD, autism, Huntington's disease, and Alzheimer's disease) was described by Xi et al. [12] and Brennan [13]. Despite that some disease phenotypes which are caused by orthologue genes can be very different, particularly when comparing fish and humans [14], it was shown that more than 70% of all human disease genes have functional homologs in *Danio rerio* [15, 16]. In this way, the fast growing and easy-to-breed zebrafish could be a reasonable choice when contemplating to keep thousands of animals at low costs [16, 17]. While zebrafish was originally considered as a bridge connection in the experimental gap between fly/worm and mouse/human in studying embryo development, it was shown that as the new research methodologies and genetic tools were updated, the zebrafish animal model was reported to be well suited to both developmental and genetic analysis [18] as well as complex human disorders [19, 20].

In this context, experimental animal models are needed to provide additional evidence on PD etiology, mechanisms, and possible therapeutic interventions. Thus, in this study, we aimed to describe the influence of OS on the Parkinsonian nervous system, as previously shown also by our research group on rodent models [12–15]. Furthermore, considering the emerging use of zebrafish in the novel worldwide research endeavours, we aimed to compare and elaborate the zebrafish neurophysiology model in PD research with regard to disease development factors, cause-effect relationship of OS and comorbidities (focussing on sleep disturbances), and the pharmacological potential of antioxidant molecules.

## 2. Materials and Methods

**Figure 2:**
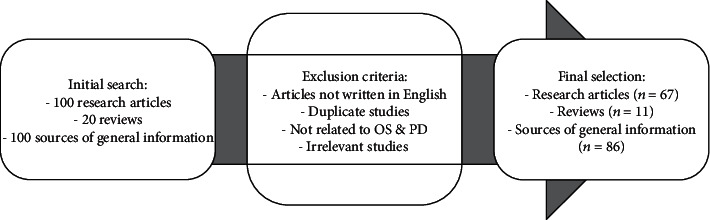
The procedure used for the selection of scientific articles.

The search strategy included the use of major scientific databases (PubMed, Google Scholar, Web of Science, and Scopus) for research of scientific articles published between 1990 and 2020. The following search keywords were used: “oxidative stress,” “Parkinson's disease,” “animal model,” “rat,” “mice,” “zebrafish,” “1-methyl-4-phenyl-1,2,3,6-tetrahydropyridine (MPTP),” “rotenone,” “paraquat,” and “neurotoxin.” The process of scientific article selection considered only reports written in English language, and the selection was conducted by four separate researchers (Robea M.-A., Balmus I.-M., Savuca A., and Ciobica A.) whose differences in opinion were resolved by common consent. After the initial scientific database research, all the scientific articles were reviewed considering some inclusion criteria, such as the reports that (1) included information/research data on the oxidative changes occurring in Parkinson's disease, or described the molecular pathways of Parkinson's disease in relation to human pathology, or presented significant results on Parkinson's disease treatments (in relation to oxidative balance pathways) and (2) included relevant information on the Parkinson's disease animal models (in relation to oxidative balance pathways), or described the molecular pathways of Parkinson's disease symptoms' means of modulation in animals, or presented significant results and correlations on the Parkinson's disease treatments (in relation to oxidative balance pathways and antioxidant potential). Exclusion criteria were formulated to avoid duplicate studies (i.e., studies on the same antioxidant molecule), studies not related to oxidative stress and Parkinson's disease pathological pathways, and irrelevant animal model studies in Parkinson's disease research (Figure 2).

## 3. Parkinson's Disease and Oxidative Stress

PD is a progressive neurodegenerative disease, which predominantly occurs in the elderly population [24–26]. Characterized by loss of neurons from the substantia nigra, PD leads to inhibition of dopamine production and accumulation of Lewy bodies (LB) formed by *α*-synuclein aggregates, a presynaptic neuronal protein [11, 27–29]. The result of these neuromolecular changes is translated into several clinical symptoms, such as bradykinesia, resting tremor, rigidity, and postural instability [27, 29–31]. Several nonmotor symptoms, such as depression, hyposmia, fatigue, sleep disturbances, and cognitive deficits such as dementia are often considered comorbidities of PD [11, 27, 29, 32].

Dopamine (DA) synthesis starts with tyrosine and two key enzymes (tyrosine hydrolase and amino acid decarboxylase), whereas its degradation is provided by the action of three key enzymes (monoamine oxidase B, catechol-O-methyl-transferase, and dopamine *β*-hydroxylase) resulting in two final metabolites and norepinephrine [14, 33]. However, excess DA induces neuronal damage and cell death through ROS generation. Furthermore, ROS accumulation could lead to DNA mutations and to loss of dopaminergic neurons from the substantia nigra [33–35].

Considering that ROS can be produced by different biological structures, many ROS functions have been described mainly according to their reactivity. Mitochondria and metabolism are some of the most important sources of ROS, thus enzymes such as nitric oxide synthase, monoamine oxidase, and xanthine oxidase produce daily huge amounts of the following reactive oxygen and nitrosative species: superoxide (O_2_^∙–^), hydroxyl (OH^∙^), peroxyl (ROO^∙^), nitric oxide (NO^∙^), nitrogen dioxide (NO_2_^∙^), dinitrogen trioxide (N_2_O_3_), nitrosonium cation (NO+), nitroxyl anion (HNO), and lipid peroxyl (LOO^∙^) [9, 10, 36, 37].

Olanow and Tatton [34] and Asanuma et al. [38] both reported an increase in lipid peroxidation and a decrease in the activity of antioxidant enzymes, such as catalase (CAT), glutathione (GSH), and glutathione peroxidase (GPx), in PD patients. Furthermore, dysregulated metal ion homeostasis has been often reported in PD development, particularly iron [39]. High iron levels were discovered in the substantia nigra pars compacta which were presumed to lead to hydroxyl radical (OH) generation due to iron's redox instability [23, 40, 41].

OS implication in PD is supported by postmortem studies and by numerous reports which suggested its active role in PD pathological processes [42]. A link between OS, mitochondrial dysfunction, and glutathione levels was suggested by Di Monte et al. [43] by the implications of DA metabolization by monoamine oxidase (MAO) during which the formation and accumulation of H_2_O_2_ occurs. Simultaneously, the glutathione resources are depleted while glutathione peroxidase catalyses the reduction of H_2_O_2_ in H_2_O [37, 38]. Furthermore, as a consequence of substantia nigra glutathione depletion and mitochondrial dysfunction, NO^∙^ production increases and leads to *α*-synuclein (*α*-syn) accumulation [10, 12, 29].

In physiological conditions, oxidised glutathione (GSSG) is reduced by glutathione reductase in reduced glutathione (GSH) using NADPH. However, the GSH depletion could be caused by impaired synthesis of GSH, which is a result of mitochondrial dysfunction since there is not an adequate quantity of ATP to sustain the GSH production [43, 44]. To support this hypothesis, Hauser et al. [45] proved that GSH is reduced approximately 40-50% in PD patients.

Similarly, an important pathological mechanism underlying PD pathogenesis could be supported by nitric oxide action due to its capacity to impair synaptic activity, memory functionality, and neuronal plasticity [34, 41]. This aspect was suggested by Ravenstijn et al. [66] while showing that 7-nitroindazole could exhibit an inhibitory effect on nitric oxide synthase from the substantia nigra pars compacta leading to malonate, 3-nitropropionic acid, or MPTP-induced lesion attenuation [46].

As we previously described, mitochondrial dysfunction plays a major role in symptom persistence and disease progression [47, 48]. The decreased rate of ATP production leads to OS and further to cell death [49]. Mitochondrial complex I is often the target in neurodegenerative PD since it plays a crucial role in the mitochondrial respiratory chain [19, 48].

## 4. Parkinson's Disease Animal Models: Rodents versus Zebrafish

Recent studies showed that mitochondrial dysfunction is a key feature of PD pathogenesis [27, 34, 41, 47, 50]. According to numerous reports, there are several chemical compounds which could influence the activity of mitochondrial complex I. In this way, the modulation of mitochondrial activity could lead to the occurrence of PD-like symptomatology in both rodent and zebrafish models. Thus, rotenone, 1-methyl-4-phenyl-1,2,3,6-tetrahydropyridine (MPTP), paraquat, 6-hydroxydopamine (6-OHDA), pyrethroids, and organophosphates [23, 51–53] were successfully used to increase the ROS levels and therefore to promote dopaminergic neuron degeneration [54]. This could be the reason why many PD animal models (mainly rodents) are based on the acute/chronic administration of some of the mentioned chemicals and also the evidence which ties OS to PD molecular pathways.  Table 1 summarises several animal model studies on PD-like impairments, chemical inductors, and comparisons between the zebrafish and rodent models.

Accumulation of *α*-syn is a clear sign of PD and one of the main causes to its development [73]. Synucleins, a family of proteins naturally occurring in the nervous system, are known to contribute to vesicle synapse maintenance or DA activity [65–68]. Zebrafish possesses three genes which encode *β*-, *γ*1-, and *γ*2-synucleins [66, 67]. Milanese et al. [14] showed that if the *β*- and *γ*1-synucleins are knocked out, zebrafish exhibits hypokinesia and low levels of DA. Zebrafish synucleins (zSynC) share a high similarity with human synucleins, wherein zSynC is 70% identical and 82% similar to human *β*-synuclein [74].

Furthermore, Prabhudesai et al. [75] suggested that *α*-syn accumulation in the zebrafish nervous system leads to neuron apoptosis and death. Their hypothesis was confirmed since CLR01, a molecular tweezer, could increase the survival rate of embryos and suppress *α*-syn aggregation in a transgenic zebrafish model carrying human wild type *α*-syn [75].

Previous studies demonstrated that overexpression of *α*-syn in a hypothalamic neuronal cell line could lead to increased ROS, mitochondrial impairment, and LB [50, 73, 76]. Also, it was suggested that cholesterol metabolites resulting from ROS degradation could promote the aggregation of *α*-syn [76].

**Figure 3:**
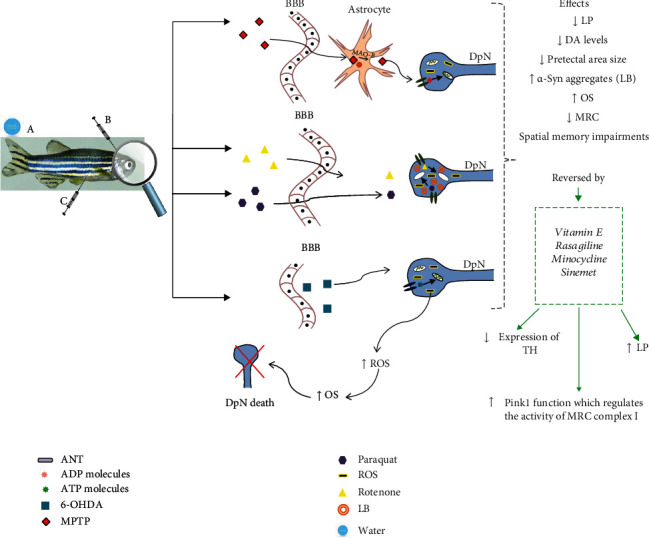
Parkinson's disease molecular mechanisms and effects in the zebrafish central nervous system induced by several Parkinson's disease agents and treatment alternatives. Administration of these chemicals (MPTP, rotenone, paraquat, and 6-OHDA) through various ways can lead to a reduction in locomotor parameter activity, a decrease of dopamine neuron number, an increase of oxidative stress, and the inhibition of mitochondrial complex I promoting the formation of Lewy body inclusions [48, 69, 70, 79]. Vitamin E, rasagiline, minocycline, and Sinemet can reverse the action of the Parkinson's disease agents mentioned above in zebrafish [80–82]. Abbreviations: ↑—increase; ↓—decrease; 6-OHDA—6-hydroxydopamine; A—dissolved in the water; ADP—adenosine diphosphate; ANT—adenine nucleotide translocase; ATP—adenosine triphosphate; B—intracerebroventricularly injection; BBB—blood-brain barrier; C—intraperitoneal injection; DA—dopamine; DpN—dopaminergic neuron; LB—Lewy bodies; L-DOPA—levodopa; LP—locomotor parameters; MPTP—1-methyl-4-phenyl-1,2,3,6-tetrahydropyridine; MAO-B—monoamine oxidase B; MRC—mitochondrial respiratory chain; OS—oxidative stress; ROS—reactive oxygen species; TH—tyrosine.

Due to several limitations of the classical chemical-inducing animal models, genetically engineered animal models are now generally preferred. However, the studies on the pathological mechanisms underlying PD development consider more appropriate the use of the chemical-inducing models, since the interplay between the dopaminergic neuron functions and brain redox activity remains a fine game of regulatory potentials. Thus, the study of OS was performed on animal models, such as *Drosophila*, zebrafish, mice, or rats, predominantly using common neurotoxicants (MPTP, 6-OHDA, rotenone, and paraquat) [23, 37, 77, 78]. Thus, the main mechanisms related to Parkinson's disease in the zebrafish central nervous system are schematically presented in Figure 3, together with several agents that induce Parkinson's disease and some treatment alternatives.

## 5. MPTP

One of the most common chemical agents which can modulate PD symptomatology in animal models is 1-methyl-4-phenyl-1,2,3,6-tetrahydropyridine (MPTP) [43, 83]. Following its monoamine oxidase B (MAO-B) promoted conversion to 1-methyl-4-phenylpyridinium in astrocytes [35, 38, 43, 56], MPP^+^ could easily bind DA transporters reaching mitochondria and interfering in the oxidative phosphorylation process carried out by mitochondrial complex I [35, 38, 44, 84]. Also, many reports described the MPTP neurotoxicity to be correlated with tyrosine hydrolase loss and DA transporters [35, 43]. Due to the fact that the participants to this mechanism are highly conserved, OS induction in this way was observed in zebrafish, mice, rats, cats, dogs, and nonhuman primates [35, 85].

In zebrafish, the reports showed that MPTP could efficiently induce decreased locomotor activity, which is caused by DA activity decrease, number of DA neurons, and pretectal size reduction [45, 49, 86–88]. Also, MPTP could induce bradykinesia manifested in zebrafish as decreased velocity and abnormal swimming behaviour [49, 86, 89]. Moreover, as compared to rodent models which seemed to overcome the short-term toxin activity on locomotion or even exhibit no changes or hyperactivity [90], zebrafish showed behavioural changes even in acute MPTP administration (Table 1).

However, neuromodulation efficiency depends on several factors, such as the administration route, sex, strain, and developmental stage [78, 91]. For example, larval zebrafish is more sensitive to MPTP than adult zebrafish [92]. Also, Jackson-Lewis et al. [93] showed that MPTP administration effects could be influencing the size of central nervous system damage in a dose-dependent manner, since they demonstrated that the impairment of tyrosine hydroxylase (TH) activity following lower doses of MPTP could not lead to DA neuron loss in mice. Thus, Kirchhoff et al. [94] reported that two MPTP injections (15 or 20 mg/kg) for 7 consecutive days were sufficient to cause dopaminergic neuron loss in mice. Thus, in a recent report of Mingazov et al. [95], it was demonstrated that 12 mg/kg b.w./day subcutaneous MPTP treatment for 2 weeks could not induce DA metabolism impairment or MAO-A and MAO-B enzyme activity changes even with DA neuron loss confirmation.

Similarly, the reports on DA neuron loss in zebrafish are rather controversial. In this way, it was shown that at 24 hours postfertilization, zebrafish embryos immersed in MPTP solution (800 *μ*M, distilled water) for 2 days exhibited posterior tuberculum DA neuron function impairment leading to behavioural deficits [96]. Also, since L-deprenyl treatment—which is a potent MAO-B inhibitor—could prevent neurodegeneration in zebrafish [55, 97], it could be suggested that a similar mammalian MPTP catabolism pathway could be present in zebrafish; however, it is a known fact that they possess a single monoamine oxidase homologous to both MAO-A and MAO-B [55].

In this way, despite the demonstrated limitations of the rodent models, mice are preferred for DA neuron loss models, since there is no previous report accounting for the neuronal loss that was not present after MPTP administration in mice and rats.

## 6. 6-OHDA

Because it is incapable of passing the blood-brain barrier, the catecholamine 6-hydroxydopamine (6-OHDA) was the first neurotoxin used to model PD [23, 98] by direct/sham injection [98, 99]. The 6-OHDA mechanism of action consists of mitochondrial complex I and IV inhibition [23, 98, 100–102] and also autoxidation resulting in O_2_^−^ and H_2_O_2_. In this way, the major effect of 6-OHDA is the DA neuron impairment.

Regarding the active potential of 6-OHDA to model PD symptomatology in zebrafish, it was observed that its administration could lead to changes in both biochemical and behavioural parameters. In this way, several studies reported that the 6-OHDA administration led to a decrease in dopamine and noradrenaline levels and also suggested that OS could develop in a cause-effect relationship with regard to 6-OHDA [40, 48]. Furthermore, it seems that 6-OHDA could successfully model in zebrafish one of the main PD symptoms—motor disturbances—as it was previously suggested that zebrafish larvae exposed to a 6-OHDA solution exhibited salient motor impairments and decreases in tyrosine hydroxylase activity [40, 48, 80–82].

Also, the other PD hallmark symptom—DA neuron loss—was evaluated in zebrafish models by Parng et al. [103] and Vijayanathan et al. [48]. Thus, they showed that irrespective of the administration route and developmental stage (e.g., Parng et al. treated zebrafish embryos with 250 *μ*M 6-OHDA dissolved in the water, while, Vijayanathan et al. microinjected 25 mg/kg 6-OHDA in the ventral diencephalon of adult zebrafish), 6-OHDA treatment as short as 3 days could lead to DA neuron loss [48, 103].

Moreover, given the many differences between zebrafish and rodents, similar effects of 6-OHDA administration were reported in rodent models [101]. Also, regarding the OS implications of 6-OHDA in rodent models, the antioxidant role of GPx overexpression and thus an increase in activity for the DA neuron protection was demonstrated. In this way, Bensadoun et al. [104] suggested that one of the most prominent components of the anti-6-OHDA neurotoxic effect is the GPx antioxidant enzyme which not only prevented the dopamine secretion inhibition but also indirectly modulated the tyrosine hydroxylase activity.

## 7. Rotenone

Despite that MPTP and 6-OHDA administrations are one of the most common ways to modulate PD symptomatology in animal models, more recent research revealed that several nonintended molecules possess the potential to induce PD [22, 23, 105]. For example, exposure to some extensively used herbicides and pesticides was shown to lead to mitochondrial dysfunction and DA neuron loss. In this category stands rotenone, which is an alkaloidal pesticide used worldwide [106] and which acts on mitochondrial complex I and DA neurons [17, 63, 106–109]. According to Alam and Schmidt's report [17], rotenone could destroy DA neurons and induce PD symptomatology in rats following 2 months of intraperitoneal treatment. Also, it was shown that rotenone administration could lead to *α*-syn aggregate formation and increased oxidant levels [15, 61, 62, 64–67].

The cytotoxic effects of rotenone seen in fish are similar to those observed in rodents. For example, the administration of rotenone in zebrafish could lead to impaired motor ability, olfactory dysfunction, and decreased DA levels [16, 110]. Also, DA neuron reduction was reported by Martel et al.'s study [111], wherein zebrafish embryos received for 7 days a dose of 30 *μ*M rotenone. These aspects were previously described to be a part of the natural mechanism of action of rotenone used to eradicate pests. Moreover, the OS-causing potential of rotenone was described and additional evidence was brought by Melo et al. [112] who demonstrated that 20 *μ*g l^−1^ rotenone can inhibit CAT, GST, and acetyl cholinesterase activity.

Another recent report showed that a 4-week administration of 2 *μ*g l^−1^ rotenone to adult zebrafish leads to 40-50% TH expression decrease. Also, the decrease of DA level was correlated with impaired locomotor activity as a motor symptom and anxiety behaviour specific to PD individuals [110]. As stated before, rotenone is a potent tool to induce motor and nonmotor symptoms of PD in a zebrafish model [111, 112].

## 8. Paraquat

Being largely used as herbicide, the paraquat mechanism of action is similar to MPTP [39]. In this way, lipid peroxidation, mitochondrial dysfunction, accumulation of *α*-syn, and low levels of GSH were reported in organism models such as rodents and zebrafish following paraquat administration [39, 70, 71, 79, 113]. Additionally, the loss of DA neurons is evidence of the effects of paraquat which can be observed in behavioural and biochemical deficits [70].

Moreover, it was shown that paraquat has the potential to increase the effect of ROS on other molecules, such as lipids, which are one of the main targets of oxidants [114, 115]. Thus, it was observed that 0.04 ppm paraquat administration to zebrafish could lead to more than a 15% increase in MDA levels after 96 hours, concomitantly with DA, GSH, and serotonin decrease [79]. Another important aspect on the paraquat mechanism of action in zebrafish models was that intraperitoneal administration could lead to increased CAT and GPx activity suggesting that its administration could boost some of the antioxidant enzyme activities in the effort of decreasing the cellular ROS levels [116].

## 9. Relevant Antioxidant Opportunities in Parkinson's Disease Treatment

Considering that healthy functioning metabolism includes a physiological anti-ROS system actively engaged in the prevention of overproduction and accumulation of ROS and that OS seems to be an important component of PD in both human and animal models, the obvious lead for a PD cure could be the antioxidant system stimulation. In this way, the antioxidant system consists of biologically active molecules responsible for ROS neutralization and cell protection against the free toxic radicals' effects [4–7]. Thus, the antioxidant enzymes, such as superoxide dismutase (SOD), glutathione peroxidase (GPx), catalase (CAT), and glutathione reductase (GRx) [10–13], and the nonenyzmatic antioxidants, such as lipoic acid, coenzyme Q10, melatonin, vitamin E, vitamin C, flavonoids, and omega acids [21], form a powerful protection system which prevents the occurrence of OS.

Regarding the implication of mitochondria in ROS metabolism, being responsible for adenosine triphosphate (ATP) production, the main chemical energy source for cellular functions [23], it was demonstrated that the reduction of the ATP synthesis and the electron transport chain impairments lead to ROS accumulation [24]. Due to the fact that mitochondria are a high-quantity ROS source, the correlation between mitochondrial dysfunction and neurodegenerative diseases was predictable. Moreover, the biochemical profile of the brain and the vital role of ROS in brain molecular signalling are further evidence that the central nervous system is continuously predisposed to OS exposure [17, 18]. Additional evidence regarding the antioxidant system and the modulatory pathways has been reported while studying antioxidant supplements, while it was observed that they could provide symptomatology relief or even to reverse oxidative changes and their effects in chronic mitochondrial diseases [24].

PD treatment is mainly based on levodopa and dopamine agonists (amantadine) [47, 114, 117, 118]. Despite the advantages of levodopa administration, dopamine agonists only partially reverse motor symptoms of PD leading to different motor oscillations. However, levodopa remains the main substance used in PD therapy [117–119].

Also, other alternatives for PD treatment are catechol-O-methyl-transferase (COMT) inhibitors and monoamine oxidase B inhibitors, such as safinamide or rasagiline [114, 120, 121]. Similarly, *α*-lipoic acid is used to remove the excess metals, and coenzyme Q10 is used to decrease oxidative marker activity [27, 114]. Another efficient therapeutic choice in PD treatment is selegine (L-deprenyl) [55, 122]. The carotenoid lycopene was described as a potent antioxidant by reducing the complex I inhibition in a rotenone rat model and reversing MPTP effects in a PD mice model [68, 123].

Besides the drug-centred therapies, exercise programs are an effective strategy used in PD patients to improve and to delay functional decline [124]. Alongside the improvement in muscle tonus, it was demonstrated that exercise leads to OS decrease and overall metabolism improvement [125], while muscle weakness is one of the main symptoms of PD, its severity increasing with time [126]. Despite that several studies reported muscle weakness as a consequence of sedentary state and aging processes, bradykinesia seems an important and independent symptom of PD; however, its occurrence mechanism is not fully understood [124, 126]. Recently, it was demonstrated that aquatic exercise therapy could improve motor disability of PD individuals [127].

Studies made until now using animal models led to new speculations regarding PD treatment and other alternatives for it. Treatment with 100 *μ*M vitamin E, 10 *μ*M minocycline, and 25 *μ*g/ml Sinemet (a well-known drug for PD which contains carbidopa and levodopa) in the presence of 25 *μ*g/ml 6-OHDA of zebrafish larvae led to a reverse of locomotor disruptions and of the changes that appeared for parkin, pink1, and cd-11b mRNA expression [80]. Locomotor deficits and neuronal loss observed in zebrafish larvae after 3 days of treatment with 250 *μ*M 6-OHDA were prevented by 10 *μ*M minocycline and 1 *μ*M rasagiline coadministration [81]. Another study also reported motor and optomotor alterations and morphological changes in zebrafish larvae induced by 250 *μ*M 6-OHDA which were ameliorated after supplementation with 1 mg l^−1^ N-acetylcysteine, which is known for its antioxidant, anti-inflammatory, and neurotrophic potential [82].

## 10. Is There an Oxidative Stress Correlation to Sleep Disturbances in Parkinson's Disease?

According to Porkka-Heiskanen et al. [128], sleep is the periodic physiological state characterized by temporal suppression of consciousness, partial loss of sensitivity, and decrease of several body functions, such as heart rhythm, respiratory rate, muscle relaxation, and body temperature. Due to its complex mechanism, many factors influence the quantity and quality of sleep, namely stress exposure, health conditions, or some forms of substance abuse [128].

Several recent studies reported that sleep disturbances are a common symptom of PD. In this way, both Menza et al. [129] and Selvaraj and Keshavamurthy [130] described that a direct correlation between PD severity and sleeping time could be suggested and assumed by memory deficits, depressive mood, body weakness, and involuntary sleep events during the day [128]. In this way, several sleep disturbances were described to occur in PD patients, such as insomnia, sleep-related respiratory disorders (SRD), excessive daytime sleepiness (EDS), and sleep-related motor problems often exhibited with variable intensities and durations [129–131]. The connection between these sleep impairments and PD could be certain mechanisms also occurring in OS, for an instance due to sleep apnea or poor oxygenation of the brain during sleeping time [132].

However, regarding the occurrence of sleep-related respiratory symptoms in PD patients, recent studies reported controversial results. In this way, sleep apnea episodes were not significantly present in PD patients, as compared to control groups [131], despite that other reports demonstrated that almost 50% of PD patients experienced sleep apnea incidents [129]. In a more recent study, Bohnen and Hu [132] reported a correlation between sleep apnea which leads to repeated periods of hypoxia and reoxygenation during sleeping and the occurrence of OS and inflammation though a similar mechanism with sleep apnea-induced chronic intermittent hypoxia models. On the other hand, other studies reported that the more common sleep disturbance in PD patients is insomnia [131, 133], which is present in 54-60% of cases [134]. However, Gjerstad et al. [134] discussed the results in the context of age, pathological lesions in the upper brainstem and midbrain, depression, nocturia, and medication [133, 135]. Thus, the multifactorial etiology of sleep disturbances [132] could be discussed in this context and also in the context of the possible comorbidity of sleep disturbances in PD. However, the cause-effect relationship between PD and sleep disturbances is not fully understood.

Another PD-occurring sleep disturbance is rapid eye movement sleep behaviour disorder characterized by motor behaviours and different vocalizations [135, 136]. By comparison with the other sleep disturbance symptoms in PD, rapid eye movement sleep behaviour disorder is being considered a premotor symptom, and in some cases a disease development marker [137] due to the fact that 40 to 65% of those diagnosed with rapid eye movement sleep behaviour disorder are further later diagnosed with PD [137–140]. Excessive daytime sleepiness (EDS) and fatigue are also present in PD [129, 131, 141, 142]. Restless legs syndrome (RLS) and periodic limb movements in sleep (PLMS) are both correlated to PD [131, 133, 141, 143, 144].

RLS is a sensorimotor condition characterized by the desire to move the legs due to unpleasant sensations [133]. Often appearing in older PD patients, RLS was correlated to iron deficiency as a secondary condition for RLS onset [133, 144, 145]. However, low substantia nigra iron levels were reported in RLS while increased iron levels in PD patients were suggested to lead to OS [142, 144, 145]. Both PD and RLS are characterized by iron deficiency, which may lead to DA damage specific for PD-RLS according to a study published in 2017 [142].

The correlation between OS and PD was previously described, but so was the idea that sleep deprivation can cause OS [146–148]. Thus, it is controversial to ask if any of these correlations could be explained in the context of the presence of all three components: PD, sleep disturbance, and OS. This aspect was partly elucidated due to animal models using certain substances or genetic manipulations for PD features. Scientific literature reported various ways to study these aspects whose target were sleep disturbances [129, 130]. Disruptions in diurnal rhythms, stress, and specific alterations in sleep architecture are only three examples of methods used in animal research [149].

In this way, a recent study presented reasonable evidence which correlated sleep disorders occurring in PD and OS. Filograna et al. [150] extensively described the mechanism through which iron chelators prevent the increase of substantia nigra iron levels in PD patients. Thus, in a well-known study, an iron-chelator-treated chronic iron-loaded mice model exhibited improved OS markers and decreased iron levels. Also, the same group described the antioxidant effects of melatonin in the context of PD-occurring OS mechanisms. The authors pointed out that melatonin antioxidant activity is not described as only free radicals scavenging, but also as other indirect modulatory activities, such as expression stimulation of several antioxidant enzymes and the downregulation of prooxidant enzymes.

Another important component of this triad is the mechanism underlying intermittent hypoxemia observed in PD patients in concomitance with obstructive sleep apnea [151]. According to Kaminska et al. [151], a potential origin of OS occurrence in PD would be the exposure to intermittent episodes of hypoxemia during the sleeping periods. It was shown that intermittent hypoxemia could lead to important changes in the brain structures involved in peripheral nerve conduction, impaired learning and memory, and neuronal loss possibly through mechanisms of ischemia/reperfusion, and oxidative injury.

The recent report of Cao et al. [152] suggested that another OS-related ion could be involved in sleep disturbances. It was demonstrated that magnesium could have long-term benefits in reducing the likelihood of falling asleep in the daytime in women, but not in men, through a mechanism that is, however, unknown. Genetic predisposition would be one of the possible responses in the matter of mechanisms.

Despite that the genetic landscape of PD is currently well described, the implication of magnesium in PD has been only recently hypothesised [153]. Recent research in human and animal models showed that low magnesium levels are correlated with increased risk to develop PD [154]. Moreover, Sturgeon et al. [154] suggested that the mechanism through which this correlation is built is based on a unique genetic landscaping of magnesium homeostasis. Sustaining this hypothesis, several studies meta-analysed by Jin et al. [155] suggested that increased magnesium levels are a molecular feature of PD, therefore magnesium dishomeostasis may be considered a real risk factor in PD. Both SLC41A1 and TRPM7 are directly or indirectly modulating sleep-related behaviours [154]. Thus, SLC41A1 is being involved in rapid eye movement sleep behaviour disorder [156], while TRPM7 is suggested to be involved in sleep-wake cycle modulation through magnesium ion ligation potential [157].

Moreover, the OS and DA perturbations were also observed in gene mutations of *α*-syn, PINK, parkin, and DJ-1 proteins [25, 52]. The lack of PINK1 leads to a loss of DA neurons, affects the mitochondrial morphology, and is linked with OS [21, 25, 158–161]. Also, the accumulation of *α*-syn causes the reduction of mitochondrial activity and a high production of ROS which is completed by cell death [25, 31]. The PARK2 and LRRK2 genes represent a source of ROS production [26]. All these proteins are linked with PD pathogenesis, and their use in genetic manipulations has become a new tool in transgenic animal models [12, 161, 162].

Thus, genetic implications in PD are not new to PD research. Several recent studies suggested not only that some genetic factors give real predisposition to PD development at some point in life [163] but also that PD may be one of the disorders which run in a family [164]. Currently, more than 20 PD genetic predisposition loci are identified and extensively reviewed [163]. Among these, some target the synaptic vesicle anomalies (SNCA mutations), the protein-to-protein interaction in the cytoskeleton assembly (dardarin gene mutations), ubiquitin degradation (parkin gene mutations), and several other energetic mechanisms.

## 11. Conclusions

This study synthesized the current information and correlated available data on the relevance of the oxidative stress status modifications in the complex pathophysiology of Parkinson's disease with regard to the available animal models. Moreover, the importance of the zebrafish model in Parkinson's disease research was described. It was observed that OS possesses an important role in Parkinson's disease as shown by numerous animal model studies, many of which are based on rodent experimental models. However, an emerging impact of the zebrafish model was observed in research on Parkinson's disease pathological mechanisms with regard to disease development factors, cause-effect relationship of oxidative stress and comorbidities (such as depression, hyposmia, fatigue, sleep disturbances, and cognitive deficits), and also regarding the pharmacological potential of antioxidant molecules in Parkinson's disease treatment.

## Figures and Tables

**Table 1:** Parkinson's disease animal models based on neuromodulation agents.

PD-inducing agent	Model organism	Treatment	Route of administration	Effects	Reference
MPTP	Zebrafish	5 *μ*g/ml and 10 *μ*g/ml, 3 days	Dissolved in the water	↓Locomotor activity ↓Pretectal area size ↓DA levels	[55]
		Single 20 mg/kg dose	Abdominal injection	↓DA and noradrenaline levels ↓Locomotor activity	[40]
		1 × 50 *μ*g and 2 × 50 *μ*g/24 h	Intraperitoneal injection	↓Locomotor activity Evidence of freezing bouts	[56]
	Rat	Single 20 *μ*l/kg dose	Intrasubstantia nigra injection	↑MDA ↓SOD	[57]
		Single 100 *μ*g/1 *μ*l dose	Bilateral infusion	↑LPO ↓GSH ↑SOD levels in the striatum	[58]
		1 *μ*mol/2 ml, in the 1st, 7th, and 14th day of the experiment	Intrasubstantia nigra injection	↑MDA ↓GSSH ↓CAT	[59]
	Mice	30 *μ*g/kg, twice at 16 h intervals	Intraperitoneal injection	↓GSH ↓SOD in substantia nigra	[60]
6-OHDA	Zebrafish	Single dose: 25 mg/kg	Abdominal injection	↓Velocity rate and locomotor activity ↓DA neurons	[48]
		Single dose: 25 mg/kg	Intraperitoneal injection	↓DA and noradrenaline levels ↓Locomotor activity	[40]
	Rats	10 *μ*g/2 *μ*l	Unilateral intrastriatal injection	↓GSH ↓CAT ↓SOD	[19]
		8 mg/2 ml	Intrastriatal injection	↑MDA levels ↓GSH and SOD levels in striatum	[20]
Rotenone	Zebrafish	1-12 mg/kg, 7 to 36 consecutive days	Intravenous injection	↓DA neurons ↑*α*-Syn aggregates	[15]
		5 *μ*g/l, 28 consecutive days	Dissolved in water	↓Locomotor activity ↓DA neurons ↑*α*-Syn aggregates	[16]
	Rats	1.5 mg/kg and 2.5 mg/kg, 2 months	Intraperitoneal injection	↓DA neurons in posterior striatum and prefrontal cortex ↑Catalepsy	[17]
		2.2-2.5 mg/kg, 28 consecutive days	Intravenous injection	↓Locomotor activity ↑*α*-Synuclein aggregates	[61]
		2.0-3.0 mg/kg, 28-56 days	Subcutaneous injection	↓DA neurons *α*-Syn aggregates	[62]
		Single 2.5 mg/kg dose	Intraperitoneal injection	↓Body weight ↓DA neurons in striatum	[63]
		2-12 *μ*g/*μ*l, 28-90 days	Stereotaxial infusion	↑*α*-Syn aggregates ↓ROS level	[64]
		2.0 mg/kg, 28 days	Subcutaneous injection	↓Locomotor activity ↓DA neurons ↑*α*-Syn aggregates	[65]
		5 *μ*g, 21 days	Stereotaxial infusion	↓DA neurons ↑*α*-Syn aggregates	[66]
		0.25-0.50 *μ*g, 21 days	Stereotaxial infusion	↑*α*-Syn aggregates	[67]
		3 mg/kg, 30 days	Intraperitoneal injection	↑MDA levels ↓GSH and SOD levels	[68]
Paraquat	Zebrafish	10 mg/kg, twice a day for 3 days	Intraperitoneal injection	↓Locomotor activity Spatial memory impairments	[69]
		1, 10, and 100 *μ*M, 4 days	Dissolved in the water	↓Mitochondrial respiration	[70]
	Mice	0, 0.89, 2.67, and 8 mg/kg, 28 days	Oral administration	↑MDA in HIP ↑Mitochondrial injury	[71]
		Paraquat (10 mg/kg) + maneb (30 mg/kg), twice a week, 9 weeks	Intraperitoneal injection	↑MDA ↑NO ↓GST	[72]

↑: increase; ↓: decrease; CAT: catalase; DA: dopamine; GPx: glutathione peroxidase; GSH: glutathione; GSSH: oxidised glutathione; GST: glutathione S-transferase; LPO: lipid hydroperoxide; MDA: malondialdehyde; NO: nitric oxide; SOD: superoxide dismutase.
